# The Impact of Heat Exposure and Sleep Restriction on Firefighters’ Work Performance and Physiology during Simulated Wildfire Suppression

**DOI:** 10.3390/ijerph14020180

**Published:** 2017-02-12

**Authors:** Grace E. Vincent, Brad Aisbett, Brianna Larsen, Nicola D. Ridgers, Rod Snow, Sally A. Ferguson

**Affiliations:** 1 Appleton Institute, School of Health, Medical and Applied Sciences, Central Queensland University, Wayville 5034, Australia; sally.ferguson@cqu.edu.au; 2 School of Exercise and Nutrition Sciences, Deakin University, Geelong 3220, Australia; brad.aisbett@deakin.edu.au (B.A.); b.larsen@griffith.edu.au (B.L.); nicky.ridgers@deakin.edu.au (N.D.R.); rod.snow@deakin.edu.au (R.S.); 3 Bushfire Co-Operative Research Centre, East Melbourne 3002, Australia; 4 Griffith Sports Physiology, School of Allied Health Sciences, Griffith University, Southport 4215, Australia

**Keywords:** firefighting, sleep restriction, physical performance, work physiology

## Abstract

This study was designed to examine the effects of ambient heat on firefighters’ physical task performance, and physiological and perceptual responses when sleep restricted during simulated wildfire conditions. Thirty firefighters were randomly allocated to the sleep restricted (*n =* 17, SR; 19 °C, 4-h sleep opportunity) or hot and sleep restricted (*n =* 13, HOT + SR; 33 °C, 4-h sleep opportunity) condition. Firefighters performed two days of simulated, intermittent, self-paced work circuits comprising six firefighting tasks. Heart rate, and core temperature were measured continuously. After each task, firefighters reported their rating of perceived exertion and thermal sensation. Effort sensation was also reported after each work circuit. Fluids were consumed ad libitum. Urine volume and urine specific gravity were analysed. Sleep was monitored using polysomnography. There were no differences between the SR and HOT + SR groups in firefighters’ physiological responses, hydration status, ratings of perceived exertion, motivation, and four of the six firefighting tasks (charged hose advance, rake, hose rolling, static hose hold). Black out hose and lateral repositioning were adversely affected in the HOT + SR group. Working in hot conditions did not appear to consistently impair firefighters work performance, physiology, and perceptual responses. Future research should determine whether such findings remain true when individual tasks are performed over longer durations.

## 1. Introduction

The combination of physical work, long working hours, and shortened sleep is commonplace for search and rescue [1], wildfire suppression [2], planned burn operations [3,4], and military deployments [5]. Such deployments can also occur in hot [6] climatic conditions, potentially placing further physiological demands on these emergency service workers. Thus, understanding how different environmental conditions impact worker productivity and safety is essential for personnel in leadership, logistics, and health and safety roles. For instance, if work-rate declines in hotter temperatures, more workers may need to be deployed or more breaks rostered, so that operational objectives can be met without individuals attempting unsustainable work-rates that may compromise their health and safety. The decisions of when, and under what conditions, more personnel are deployed should be based on a robust evidence-base [7].

The existing literature investigating the impact of sleep restriction in the heat has focused on impairments in temperature regulation and thermal strain [8,9,10]. While slight impairments in temperature regulation were reported [8,9], translating these findings to an emergency response work environment is difficult. For example, these studies employed modes of exercise, such as cycling and running, over short durations (30–45 min) which is very different to the manual handling work performed over hours or days by emergency service workers [11]. Furthermore, these studies do not report measures of physical work output. As such, it cannot be determined whether thermoregulatory impairments translate into meaningful differences in work productivity and if this places workers at increased risk.

In Australia, wildfires can occur in both hot and temperate conditions, with ambient temperatures ranging from 16–45 °C [12,13]. The existing literature suggests that, in the main, wildland firefighters are capable of performing physical work without excess thermal strain or exertion [6,14,15]. However, there have also been numerous documented cases of heat-related illness and, in some instances, death on the fireground [16,17,18]. To date, the impact of these temperatures on work performance within a wildfire environment has not been quantified due to the inherent challenges in measuring work performance in variable unpredictable environmental conditions. Recently, a study simulating a 6-h simulated wildfire shift (utilising a high fidelity wildfire suppression simulation, and a between-subjects design) noted that, with unlimited access to fluids, firefighters performing self-paced firefighting work in hot conditions (32 °C, 43% RH) could be equally effective (no difference in physical task performance) as those in temperate conditions (19°C, 56% RH) [19]. Furthermore, research utilising the same simulation over three-consecutive days, conducted either in temperate (19 °C) or hot (32 °C) conditions, or with 8-h and 4-h sleep opportunities, reported no difference in firefighters’ work output across a range of firefighting tasks [20,21]. However, no studies to date have examined the combined impact of high ambient temperatures and restricted sleep in firefighting. Such studies will provide important insights into firefighters’ work performance in common working conditions, which may be important in preserving the health and safety of fire personnel.

In order to provide data that will support operations where sleep is truncated over several days of hot temperatures requires a specific experimental design. Specifically, consecutive days of work in either hot or temperate conditions, with each day separated by restricted sleep at night are needed. Therefore, the aim of the current study was to compare physical work output (physical work performance) and associated indices of work physiology (e.g., heart rate, core temperature, hydration, rating of perceived exertion, and thermal sensation), during two successive 10-h shifts following consecutive nights of 4-h sleep restriction. One group worked in temperate conditions (19 °C) while the other worked in hot conditions (33 °C). In the absence of previous literature, the authors tentatively predicted that performance of simulated firefighting work would be adversely impacted with the combined impact of heat exposure and sleep restriction, and that this decrease in work productivity would regulate physiological and subjective responses.

## 2. Materials and Methods

### 2.1. Participants and Screening

Thirty volunteer and career firefighters (27 males, 3 females) from state fire agencies in Southern Australia (Victoria, South Australia, New South Wales, Tasmania) participated in this study. All data were collected during the autumn and winter months (i.e., outside of peak wildfire season). Firefighters from hotter climates (e.g., Northern Australia) were excluded from the study to control for the potential confounding variable of heat acclimation. To reduce variation between experimental groups, participants were matched-paired based on sex, age, and body mass index (BMI), and then one of each pair was randomly allocated to the sleep restriction condition (SR) or the hot and sleep restriction condition (HOT + SR) and the other member of the “pair” allocated to the other condition. The last HOT + SR group (*n =* 4) were unable to be tested due to unavoidable operational time and cost constraints, resulting in uneven participant numbers between groups. As such, final participant numbers were *n =* 17 in the SR group, and *n =* 13 in the HOT + SR group. Participants were tested in groups of three to five. A between-subjects design was employed as the time that firefighters were required to give up to participate in this investigation (five days without remuneration) prevented a within-subjects design.

Participants provided written informed consent and completed a general health questionnaire [22] to ensure they were healthy. They were excluded if they had any contraindications to exercise, and or a diagnosed sleep disorders (e.g., sleep apnea). Participants were also asked to report their habitual caffeine consumption and physical activity levels. Participant characteristics are shown in Table 1. Ethical approval was obtained from the Deakin University Human Research Ethics Committee (DUHREC 2014-040) and the Human Research Ethics Committee of Central Queensland University (H12/01-016).

Participants were instructed to maintain their normal sleep behaviour in the lead up to the study and wore an objective monitor (Actical MiniMitter/Respironics, Bend, OR, USA) for two days prior to the commencement of the study. The Actical indirectly assesses sleep by sensing motor activity at the wrist and uses validated algorithms to distinguish sleep from wakefulness [23]. Participants completed a sleep diary and wore the Actical on their non-dominant wrist to evaluate sleep hours and timing of sleep periods. Data were sampled in 1-min epochs, with a sensitivity of <40 counts per epoch to distinguish between sleep and wake states [24].

Prior to testing, participants’ height was measured (by trained research staff) without shoes using a stadiometer (Fitness Assist, Wrexham, UK), and semi-nude body mass (i.e., underwear only) was measured using a calibrated electronic scale (A and D, Japan). Participants wore their own firefighting personal protective clothing throughout the simulation. This included a two-piece jacket and trouser set made from Proban^®^ cotton fabric (Protex^®^, Melbourne, Australia), suspenders, boots, gloves, helmet, and goggles (total weight amounting to ~5 kg).

### 2.2. Experimental Protocol

**Figure 1:**
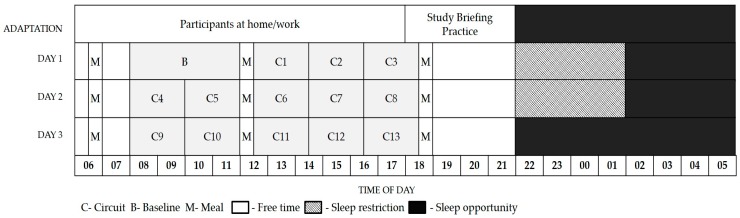
Schematic of study protocol.

The protocol lasted for four consecutive days, and participants were blinded to the study condition allocation (SR or HOT + SR) prior to arrival (see Figure 1 for overview of experimental protocol). Participants arrived at the testing facility at 6:00 p.m. on the day before the trial and were briefed on the daily procedures. Throughout the testing period, participants followed a strict daily schedule including work circuits, meal times, and sleep periods. The simulated workday comprised multiple 2-h work circuits, with each circuit consisting of 55 min of physical work, 20–25 min of physiological testing, 20–25 min of cognitive testing, and a 15–20 min rest period. The suite of cognitive procedures were conducted as part of another study and will be reported elsewhere. Caffeine consumption and cigarette smoking could continue as they normally would during a wildfire suppression deployment, but these behaviours were restricted to the rest periods (last 15–20 min of the 2-h work shift). Firefighters’ smoking status (non-smoker, ex-smoker, regular smoker) was distributed evenly between the groups.

For the SR condition a temperate ambient temperature was selected (19 °C, 40% RH) for both day-time and night-time temperatures. For the HOT + SR condition a moderately hot day-time ambient temperature was selected (33 °C, 40% RH) and night-time temperatures were maintained at 25 °C, 40% RH, to simulate the often warm sleeping conditions experienced by firefighters who sleep in temporary accommodation at (or near) the fireground [7,25]. Ambient air temperature was maintained throughout the simulated work shifts through the use of split cycle air-conditioners (Daikin Industries Ltd., Osaka, Japan) and portable ceramic disk heaters (Micro Furnace, Sunbeam, Adelaide, Australia), and was measured and monitored continuously using a wireless temperature and humidity data logger, data receiver, and associated software (HOBO^®^ Pro Software, One Temp Pty Ltd., Marleston, Australia).

To replicate sleeping conditions during a wildfire suppression deployment [2,25], participants slept on camp beds in the simulated environment. The arrival day (day 0) involved an adaptation night (night 1) consisting of an 8-h sleep opportunity for both conditions. On the following day (day 1) firefighters in each condition were thoroughly familiarised with the physical work, cognitive testing, and physiological procedures by performing three 2-h work circuits (12:30–2:30 p.m., 2:30–4:30 p.m., and 4:30–6:30 p.m.) in each group’s respective conditions (aforementioned ambient temperatures). Prior to the evening of day 1, both conditions were assigned a 4-h sleep opportunity from 2:00 a.m. to 6:00 a.m. (night 1 and night 2). Participants were constantly observed by research personnel to prevent sleep outside the designated sleeping periods. Between dinner (7:00 p.m.) and the beginning of the sleep opportunity (2:00 a.m.), participants engaged in sedentary leisure activities (i.e., reading, watching television). On both days 2 and 3, firefighters performed five 2-h work circuits (8:00–10:00 a.m., 10:00 a.m.–12:00 p.m., 12:30–2:30 p.m., 2:30–4:30 p.m., and 4:30–6:30 p.m.). Therefore, all circuits performed after the baseline circuits were labelled, 1–10.

Daily fluid consumption was recorded to measure participants’ self-selected fluid intake. Participants were able to drink room temperature water ad libitum from supplied bottles. Each day, participants were provided with two sachets of carbohydrate-electrolyte supplement which they could add to their water at any time. Breakfast (6:30–7:00 a.m.), lunch (12:00–12:30 p.m.), and dinner (6:30–7:00 p.m.) times were the same on all days, and items were standardised and based on food typically available to firefighters during wildfire suppression. Likewise, participants were also provided with a “ration pack” containing a variety of snack items similar to those provided during wildfire suppression. All meal, snack, and ration pack food items were selected in consultation with subject matter experts from Australasian fire authorities. The types and quantities of ingested food and fluid were recorded using the FoodWorks 7 nutrition software (2012 Xyris Software Pty Ltd., Australia).

### 2.3. The Physical Work Circuit

The firefighting circuit was developed using a job task analysis of wildfire suppression tasks [11], and was verified by panels of firefighter subject matter experts [26]. The tasks simulated the actions, fitness components, and movements performed during wildfire suppression in the field [11]. These tasks were chosen on the basis of being the longest, most intense, or most frequent tasks performed during wildfire suppression work [27]. The selected tasks were also considered to be the most physically demanding and operationally important [11]. The six physical tasks were: charged hose advance, black out hose work, hose rolling, lateral repositioning, rake, and static hose hold. Further details on the equipment and specific work to rest ratios used for each task are provided in Table 2.

The 55-min work circuit was broken down into 5-min increments. Within each 5-min increment, task-specific work-to-rest ratios were employed based on the ratios observed during live fire suppression conditions [27]. Some tasks were only performed once in each 55-min circuit (e.g., for five minutes), whereas others were performed more than once according to their recorded frequency during live fire suppression [27]. Participants completed the tasks in an ordered circuit. To allow multiple participants to be tested at once, each participant began the circuit at a different task station (charged hose advance, blackout hose work, hose rolling, lateral repositioning, or rake), but rotated through each subsequent task in the same order irrespective of start point. The static hose hold task was the last task performed in the circuit, and by all participants concurrently. SR and HOT + SR participants were randomly allocated to a circuit starting point. This process prevented potential clustering of specific demographic variables into any one of the five circuit start points. Each participant began at the same individually-assigned start point during all physical work circuits. The number of repetitions completed were recorded and then converted to a distance (m), or in the case of the rake task, an area (m^2^).

### 2.4. Physiological Measures: Heart Rate, Core Temperature, Hydration, and Polysomnography

In brief, heart rate and core temperature were recorded continuously using the Team Polar (Polar Team^2^, Kempele, Finland) heart rate monitor and VitalSense (Minimitter, Bend, Oregon) system. All urine was measured for volume, and Urine Specific Gravity (USG) was analysed using a portable refractometer (Atago, Tokyo, Japan). Participants were considered hydrated if their USG values were <1.020, in accordance with standard criteria [29]. Polysomnography recordings were set up and scored (by a blinded scorer) according to standard procedures and criteria [30]. From each sleep period, participants’ total sleep time was calculated. These measures have been described in further detail elsewhere [20,21].

### 2.5. Perceptual Responses

Participants reported a rating of perceived exertion (RPE, on a scale of 6–20) and thermal sensation (on a scale of 0–8) after each work bout (i.e., every 5 min) [31,32]. After the completion of each physical work circuit, participants were asked to report their perception of effort as a percentage of their maximal effort (i.e., 0%–100%) [33]. Participants were asked “how much of yourself did you give”? Items ranged from 0% “gave no effort at all” to 100% “gave absolutely everything, nothing left”.

### 2.6. Statistical Analyses

All statistical analyses were completed using Stata 12.0 (StataCorp, College Station, TX, USA). Exploratory data analyses were conducted to determine whether these data met parametric assumptions of normality and homoscedasticity. Participant characteristics at baseline, as well as total sleep hours, energy intake, hydration status, and caffeine consumption, were normally distributed. One-way analyses of variance (ANOVA) were used to determine between-group differences. For all other outcome variables, generalised linear mixed models (GLMMs) were constructed using the Generalized Linear Latent and Mixed Model software (*gllamm*; version 2.3.20, University of Berkley, Berkeley, CA, USA). This modelling procedure has been previously employed in sleep literature [34] and is increasingly preferred over traditional repeated-measures ANOVA, as GLMMs better account for the serial correlation of data points over time [35].

The framework recommended by Singer [36] guided the construction of mixed models to iteratively investigate the fixed effects of Condition and Circuit, and the interaction of Condition × Circuit, with random intercepts and random slopes that varied at the Participant-level. The model structure was condition (Level 1) and participant (Level 2). As participants were matched for age, sex, BMI a priori, these were not included as covariates in the models. Due to the differences in caffeine and energy consumption between the two conditions, these were included as covariates in all models. Differences in work performance were noted on some tasks after the 6-h familiarisation shift, therefore task performance on each task were included as a covariate for the respective models investigating work performance across the simulation. All dependent variables exhibited a normal distribution, and thus the identity link function was specified. The SR and HOT + SR conditions were coded 0 and 1, respectively. Therefore, a positive β value for the effect of Condition indicates HOT + SR > SR, and a negative β value indicates SR > HOT + SR. For Circuit effects, a positive β value indicates an increase over successive circuits, whereas a negative β value indicates a decrease. The random effects express the variance due to the inter-individual differences at baseline (random intercept) and over time (random effect of Circuit). Selection of the optimal model for each outcome variable was informed by comparing Akaike weights between candidate models. If the Akaike weights between two competing models had similar probabilities of being the best model, the model with the fewest number of parameters was preferred in accordance with the principle of parsimony. The final parameter estimates are reported in-text as β coefficient ± standard error of the estimate (SE), *p* value. Statistical significance was set at *p* ≤ 0.05 and all data are presented as means ± standard deviations, unless otherwise stated.

## 3. Results

There were no significant differences between the SR and HOT + SR groups for participants’ age, weight, height, BMI, firefighting service, and self-reported habitual caffeine consumption or physical activity (*p* ≥ 0.123; Table 1). Those in the HOT + SR group consumed on average more energy per day (15,233 ± 2054 kJ) than the SR group (13,201 ± 2850 kJ; *p* < 0.001). However, those in the SR group consumed more caffeine on average per day (184 ± 143 mg) than the HOT + SR group (120 ± 106 mg; *p* = 0.010). Mean sleep duration during the two days preceding the simulation (SR 6.4 ± 1.0 h; HOT + SR 6.9 ± 0.7 h; *p =* 0.194) and during the adaptation night (SR 6.4 ± 0.7 h; HOT + SR 6.3 ± 1.2 h; *p =* 0.414) were not different between groups. During the two nights of sleep restriction, mean sleep duration was also not different between the groups (SR 3.6 ± 0.3 h; HOT + SR 3.5 ± 0.5 h; *p ≥* 0.213). Room temperature (SR 19.6 ± 1.6 °C; HOT + SR 32.7 ± 2.3 °C; *p* ≤ 0.001) and relative humidity (SR 55.9% ± 6.8%; HOT + SR = 43.3% ± 4.0%; *p =* 0.037) were significantly different for the two experimental groups.

### 3.1. Physical Task Performance

Raw physical task performance data (daily mean performance) are reported in Table 3. On day 1 (prior to the sleep restriction intervention) participants in the SR group performed more repetitions on the black out hose (β = −18.69 ± 5.28; *p* < 0.001), lateral repositioning (β = −70.23 ± 19.62; *p* < 0.001) and charged hose advance tasks (β = −10.29 ± 4.88; *p* = 0.035), compared to the SR + HOT group. The Circuit (time) model best explained the variance in the hose rolling and rake tasks (*p* ≤ 0.013), which demonstrates that participants performed more repetitions on these tasks as the 6-h shift progressed, irrespective of Condition. All participants in both conditions completed the 5-min static hose hold task.

On day 2 and 3, all participants in both conditions completed the 5-min static hold hose task during every 2-h work period, and therefore these data will not be reported. Participants in the SR condition performed more repetitions on the black out hose (β = −10.93 ± 4.37; *p* = 0.012) and lateral repositioning (β = −40.83 ± 14.38; *p* = 0.005) compared to the HOT + SR group. Therefore, those in the SR group covered on average 10.93 m and 40.83 m more on these tasks, respectively, compared to the HOT + SR condition (*p* < 0.001). For the hose rolling, rake, charged hose advance tasks, random-slopes models best explained the variance in physical task performance (*p* < 0.001); the fixed effect of Condition did not significantly improve upon the amount of variance (*p* ≥ 0.300).

### 3.2. Heart Rate

Average heart rate data (daily mean per task) are reported in Table 2. On day 1, average heart rate per task was best explained by random slope models (*p* ≤ 0.004) for almost all tasks, highlighting a large amount of individual variation. Average heart rate during static hose hold was best explained by the conditional growth model with random slopes (β = 10.08 ± 3.92; *p* = 0.010), meaning those in the HOT + SR had elevated heart rates when compared to the SR group for this task.

On days 2 and 3, for all tasks, the Circuit (time) only model best explained the variance in heart rate (β = −0.37 ± 0.09; *p* ≤ 0.004). However, the parameter estimates across all tasks was very small (0.4% of maximal heart rate) and therefore does not likely reflect a meaningful decrease in heart rate as the simulation progressed. The fixed effect of Condition did not explain a significant amount of the variance in firefighters’ heart rate (*p* ≥ 0.570).

### 3.3. Core Temperature and Hydration

Core temperature and hydration data are reported in Table 4. On day 1, core temperature was on average 0.24 ± 0.05 °C higher in the HOT + SR group compared to the SR group (*p* < 0.001). There were no differences in USG (*p* = 0.149) or urine output (*p* = 0.600), despite greater fluid intake in the HOT + SR group (*p* < 0.001). Furthermore, prior to starting work on day 2 (6:30 a.m.–8:00 a.m.) there were no differences in USG (*p =* 0.416) and both groups were considered hydrated (SR 1.012 ± 0.007; HOT + SR 1.009 ± 0.005).

On days 2 and 3, core temperature across the simulation was best explained by the random slopes model (*p* < 0.001) with no difference in average core temperature between conditions (*p =* 0.186). The variance in daily fluid consumption was best explained by the conditional growth model with random slopes (β = 2950 ± 566; *p* < 0.001), suggesting that participants in the HOT + SR consumed more fluid than the SR group. The variance in urine output volume and USG were best explained by the random slope model (*p* < 0.001). The fixed effect of Condition did not significantly improve upon the amount of variance (*p* ≥ 0.311).

### 3.4. Perceptual Responses

All mean daily RPE and thermal sensation data are reported in Table 3. Daily mean effort sensation data are reported in Table 4. On day 1, random-slope models best explained the most amount of variance in ratings of perceived exertion (*p* < 0.001) and effort sensation (*p* < 0.001); there was no effect of Condition (*p* ≥ 0.137). Thermal sensation for all tasks was best explained by the conditional growth model with random slopes (*p* ≤ 0.039). The average parameter estimate across all six tasks was 0.94, which equates approximately to the difference between “Warm” and “Hot” on the thermal sensation scale [32].

On days 2 and 3 there was no difference in effort sensation between conditions (*p* = 0.879). The Circuit (time) only model best explained rating of perceived exertion scores for the black out hose (β = 0.06 ± 0.02; *p* = 0.001), charged hose advance (β = 0.14 ± 0.31; *p* < 0.001), lateral repositioning (β = 0.05 ± 0.01; *p* < 0.001), hose rolling (β = 0.13 ± 0.02; *p* < 0.001), and static hose hold (β = 0.06 ± 0.03; *p* = 0.045). These results demonstrate that participants reported higher RPE values as the simulation progressed, irrespective of condition. Random-slope models best explained the most amount of variance for RPE during the rake task (*p* < 0.001). The conditional growth model with random slopes best explained the variance in thermal sensation for black out hose, charged hose advance, lateral repositioning, hose rolling, and static hose hold (*p* ≤ 0.037). For the rake task, the conditional growth model best explained the variance in thermal sensation (*p =* 0.001). Therefore, participants in the HOT + SR group felt hotter when performing all tasks compared to the SR group.

## 4. Discussion

This study is the first to examine the impact of two consecutive nights of sleep restriction while performing physical work, in both temperate and hot ambient temperatures, on work performance, physiology, and perceptual responses. Partially supporting our hypothesis, the combination of heat and sleep restriction slightly reduced work performance on two wildland firefighting tasks (black out hose and lateral repositioning). Firefighters’ fluid intake was greater in the HOT + SR group, though both groups were considered hydrated throughout the simulation (according to USG values). This indicated that firefighters were able to adapt their drinking behaviour to avoid dehydration. It is also likely that participants in the HOT + SR group consumed more fluids due to an increased sweat rate, although sweat rate was not measured in the current study. No differences in core temperature and heart rate were observed. As expected, participants reported feeling hotter in the HOT + SR group, though interestingly there was no difference in RPE or motivation (effort sensation) compared to the SR group. These findings suggest that when sleep restricted and working in hot conditions, firefighters may pace themselves to match their physiological workload (heart rate and core temperature) by slightly decreasing work performance on some tasks.

When performing physical work under conditions of sleep restriction, hot ambient temperatures differentially impacted firefighters’ work performance when compared to the temperate environment. There were no differences between conditions for the rake, hose rolling, charged hose advance, and static hose hold tasks. However, firefighters’ working in temperate conditions covered on average 10 m and 41 m more per circuit, respectively, during the black out hose and lateral repositioning tasks when compared to those in hot conditions. Out of all six tasks, black out hose and lateral repositioning involved the greatest amount of walking (see Table 3 for distance averages). It is possible that, because these two tasks were of a lower intensity (as reflected by lower heart rate values; Table 3), the firefighters in the hot condition may have been pacing themselves by walking slowly in order to maintain productivity during the higher intensity fire suppression tasks (e.g., the rake task). Previous research utilising the same wildfire simulation observed no difference in firefighters’ physical task performance in conditions of 19 °C vs. 33 °C with 8 h sleep opportunity [20], nor between 8-h vs. 4-h sleep opportunity in 19 °C [21] over three days. Therefore, it appears that for certain tasks, the combined impact of sleep restriction and heat exposure may be cumulative, thus leading to a greater decrease in work performance than either stressor in isolation. As a result, worker productivity on tasks that involve walking (or low-intensity work) may need to be considered by fire agencies when workers are sleep restricted and operating in hot weather conditions. While the difference in work performance between these tasks appear small, larger differences in work performance may become apparent when tasks are performed for longer durations. In the current study, while the work to rest ratios of the tasks were reflective of those during real wildfire suppression [11], tasks were compressed into 55-minute work circuits. During real wildfire suppression, the tasks may be performed for a substantially longer time which could exacerbate the observed changes in work performance. Further, the frequent rest breaks and task rotation may have buffered possible decrements of heat on work performance. Indeed, others have reported certain firefighting tasks being performed over a wide range of durations (e.g., 4 s–7.7 min) [27]. Therefore, future studies should investigate the impact of firefighters performing single tasks extending over longer durations (e.g., >5 min) under hot conditions on self-paced physical work performance.

Unexpectedly, differences in work performance were noted between conditions during the 6-h familiarisation simulated wildfire shift prior to the sleep restriction intervention (day 1). Firefighters working in temperate conditions covered on average 19 m, 70 m, and 10 m more ground on the black out hose task, lateral repositioning task, and charged hose advance task, respectively, compared to those working in hot conditions. These results were in contrast to a previous study utilising the same wildfire suppression simulation, comparing the same ambient temperatures, albeit with a different group of firefighters [19]. Larsen and colleagues (2015) observed no decrements in work performance in all tasks, apart from lateral repositioning where firefighters working in temperature conditions covered 31 m more than firefighters working in hot conditions. The inconsistencies between the current study and that of Larsen and colleagues (2015) highlights the need for further research before consensus can be reached regarding heat and manual-handling work performance. To control for the impact of the potential differences in work performance during the 6-h shift between groups, performance was included as a covariate into work performance models post the sleep intervention. However, it should also be noted that differences in heart rate and core temperature were relatively small during this 6-h shift, and thus unlikely to impact firefighters’ next day performance.

Previous research has observed higher heart rates when performing manual handling work in the heat when compared to temperate conditions (albeit, participants were well-rested and not sleep restricted) [37,38]. However, in the current study, no differences were detected in heart rate between conditions. Given that there were no differences in task specific heart rate (despite a decrease in work performance in the black out and lateral repositioning task), it is possible that firefighters paced themselves to match their cardiovascular response by decreasing their work output [39].

There were no differences in core temperature between conditions across the simulation. In our previous work utilising hot conditions and sleep restriction protocols in isolation, small but significant increases (0.24 °C and 0.15 °C, respectively) in core temperature were observed [20,21]. Given that these two stressors were combined in the current study, and the heat load was compensable (as core temperature was observed to plateau) [40], the typical core temperature differences during exercise in the heat could have been negated by the “baseline” sleep restriction. Indeed, impairments to temperature regulation have been noted in previous studies that prescribed more severe levels of sleep deprivation (27–33 h of extended wakefulness) in hot conditions [8,9]. However, it is important to note that work performance on some tasks was different prior to sleep restriction (during the 6-h familiarisation shift following an 8-h adaptation sleep), and core temperature was 0.24 °C hotter in the HOT + SR group compared to the SR group during this time. It is also likely that, because the SR group performed more work than the HOT + SR group on some tasks, this may have resulted in a greater increase in core temperature as a result of enhanced metabolic heat production [41]. Finally, the regular rest periods employed in this study, as well as other adaptive behaviours (e.g., removal of jackets during rest breaks), may have allowed participants to maintain their core body temperature across the simulation. Whatever the explanation, these findings indicate that firefighters were not under additional thermal strain whilst working in hot conditions while sleep restricted, which is a positive outcome for fire agencies.

As with previous work in hot conditions [19], perceptual measures of thermal sensation were as expected; participants in the HOT + SR group felt hotter than the SR group. However, unlike our previous work in hot or sleep restricted conditions [20,21] , participants in the current study reported greater levels of perceived exertion, irrespective of condition, as the simulation progressed. It is worth nothing that the average increase in rating of perceived exertion was 0.1 units, which on a 6–20 point scale is unlikely to reflect a meaningful increase across time. Unlike previous work, which suggested that high core temperatures reduced the motivation for physical effort [42], the addition of heat in the current study appeared to have no impact on motivation (effort sensation). It is possible that working in groups (three to five firefighters) and constant researcher monitoring may have mitigated declines in participant motivation.

Both groups were equally hydrated throughout the simulation, highlighting the ability of firefighters to adequately regulate their fluid intake in different ambient temperatures, even when sleep restricted. In order to achieve this, the HOT + SR increased their fluid intake by drinking more water. Similar urine output values, in spite of the increased fluid intake, indicates that the sweat output of firefighters in the HOT + SR group was greater, which is not unexpected given that evaporation of sweat (in conjunction with cutaneous vasodilation) reflects the primary heat dissipation mechanism during environmental and exercise heat stress [43]. This could have also contributed to the lack of difference in observed core temperature, as adequate fluid replacement can blunt increases in core temperature [44]. While the study design precluded the accurate measurement of whole body sweat loss via changes in body mass, future studies should use the ventilated capsule or technical absorbent method to verify that the observed similarities in core temperature response resulted from increases in sweat rate (permitted by greater fluid intake) in the heat.

To further elucidate why work performance was reduced in some tasks but not others, future studies could measure firefighters task specific energy expenditure (e.g., by using indirect calorimetry). Such approaches could enable further understanding of the metabolic heat load of each task. However, the benefits of this novel step forward would need to be weighed against the significant increase in individual participant burden, reduction in ecological validity, and substantial increase in research time and cost to measure energy expenditure across multiple participants in a team environment such as firefighting. Further, the between-subjects design of this study likely contributed to the large amount of inter-individual variation in work performance observed. Therefore, future studies should implement within-subject designs to reduce individual differences in work performance. It is also important to note that the findings of the current study should not be extrapolated to firefighting in more extreme ambient temperatures (>33 °C).

The current study enabled us to quantify the effects of sleep restriction and ambient temperature on firefighters’ physical task performance by implementing a high-fidelity wildfire simulation. However, it is possible that this artificial environment was not entirely representative of real wildfire suppression; for example, radiant heat (e.g., from the fire), wind speed, steep terrain, and smoke exposure represent stressor conditions potentially encountered in the field. It is possible that the inclusion of these additional stressors in a real wildfire scenario may increase thermal stress and motivation, which could eventually result in a decline in firefighters’ physical work performance.

## 5. Conclusions

The current study found that when working under hot or temperate ambient temperatures for two consecutive days while sleep restricted, firefighters’ physiological responses, hydration status, ratings of perceived exertion, and motivation were not statistically different. Conversely, the two simulated work tasks that required the most locomotion were adversely affected in the heat, albeit to a relatively small degree. Future research should continue to investigate the combined impact of stressors when individual tasks are performed over longer durations (>5 min) and when protocols are extended over prolonged durations (>2 days).

## Figures and Tables

**Table 1:** Characteristics of firefighters in the SR and HOT + SR conditions.

	SR	HOT + SR
*N*	17	13
Age (y)	39 ± 15	41 ± 17
Weight (kg)	93.8 ± 20.2	83.8 ± 14.3
Height (m)	1.78 ± 0.07	1.76 ± 0.04
BMI (kg·m^−2^)	29.6 ± 5.5	27.0 ± 4.3
Service (y)	10 ± 6	14 ± 12
Males: Females	15:2	12:1
Habitual daily caffeine intake (mg)	150 ± 80	155 ± 83
Habitual weekly physical activity (sessions per week)	2.4 ± 2.2	3.0 ± 2.9

All data are reported as means ± SD. BMI, body mass index.

**Table 2:** Physical work circuit tasks.

Task	Parameters	Work to Rest Ratio	Times Completed	Performance Measure	Simulates
Rake	Rake the contents of a box (2 m × 0.8 m) filled with 29 kg of large and small tyre crumbs from one side to the other using a rake-hoe.	90 s work 60 s rest 90 s work	1	Number of completed boxes raked (movement of material from one side to the other).	Clearing ground debris to create a mineral earth firebreak [28]
Lateral repositioning	Hold a weighted hose (length 3.5 m) and walk in an 11-m arc. Two platforms (68 × 28 × 15 cm) served as obstacles. One platform was required to be stepped on, the other stepped over.	30 s work 30 s rest × 4	4	Number of semi circles completed (later converted to distance covered).	Moving a charged hose sideways from a fixed point, whilst negotiating fireground debris (e.g., fallen logs, tree roots; [11]).
Hose rolling	Roll up a 16-m (folded in half to a length of 8 m) hose to an operational standard. Must be rolled along the ground. Firefighter must move along the length of the hose rather than pulling towards them.	60 s work 60 s rest 60 s work	1	Absolute number of hoses rolled during the allocated work period.	Rolling up a hose for storage [11].
Charged hose advance	Drag a 2-m weighted hose attached to a 15-kg weighted tyre up and down a marked distance of 8 m.	65 s work 55 s rest 65 s work	1	Absolute number of completed hose drags from one end of the 8-m line to the other.	Walking forward with hose filled with pressurised liquid towards a fire front [11].
Black out hose work	Drag a 2-m weighted hose connected to a 15-kg bag around the perimeter of a 10-m square (2.5 × 2.5 m) pausing for 3 s at each corner (timed by a metronome).	90 s work 60 s rest 90 s work	2	Number of full rotations around the square (later converted to distance covered).	The stop-start movements that firefighters perform when extinguishing smouldering debris, during post-fire clean up [11].

**Table 3:** Daily mean work performance, heart rate, RPE, and thermal sensation for the sleep restriction (SR) and hot and sleep restriction (HOT + SR) conditions.

Task			Work Performance (m or m^2^)			Average Heart Rate (% of HR Max)			RPE			Thermal Sensation		
			Day			Day			Day			Day		
			1	2	3	1	2	3	1	2	3	1	2	3
Black out hose	SR	Mean	167.9	167.0	167.4	67	62	62	12.0	12.3	12.3	4.5	4.7	4.7
		SD	16.0	17.1	18.6	7	5	7	1.3	1.2	1.2	0.8	0.7	0.6
	HOT + SR	Mean	149.4	139.3	140.2	71	63	61	12.0	11.9	12.1	5.4	5.3	5.6
		SD	17.8	19.4	15.4	12	9	8	1.6	1.9	1.8	0.9	1.0	0.9
Charged hose advance	SR	Mean	99.9	104.4	108.0	75	70	70	14.2	14.3	14.5	5.4	5.5	5.3
		SD	12.3	16.1	17.6	8	7	8	2.1	1.7	1.8	1.0	1.0	0.9
	HOT + SR	Mean	91.3	88.9	89.9	82	73	70	15.3	14.7	15.2	6.0	5.9	6.1
		SD	18.2	19.5	19.8	13	11	10	2.0	2.1	2.3	0.7	0.8	0.9
Lateral hose repositioning	SR	Mean	650.5	656.5	679.7	67	62	63	11.2	11.4	11.4	4.3	4.5	4.6
		SD	76.7	76.8	73.3	7	6	7	0.9	1.0	1.0	0.7	0.5	0.5
	HOT + SR	Mean	573.8	560.7	550.6	70	63	61	10.8	11.1	11.9	5.2	5.2	5.5
		SD	59.1	69.2	67.1	11	9	8	0.8	0.7	0.9	0.8	0.8	0.7
Hose rolling	SR	Mean	16.9	19.0	20.5	68	63	64	11.6	12.1	12.4	4.3	4.5	4.6
		SD	3.6	3.9	5.0	8	7	8	1.3	1.0	1.0	0.7	0.6	0.6
	HOT + SR	Mean	17.4	19.5	19.9	72	65	63	11.9	11.9	12.8	5.3	5.4	5.7
		SD	4.5	6.0	5.6	12	10	9	2.3	1.7	1.9	1.0	0.9	0.8
Rake	SR	Mean	4.6	4.9	5.1	75	69	70	14.0	14.0	14.0	5.2	5.3	5.2
		SD	0.6	0.8	0.8	8	6	8	1.7	1.2	1.3	0.8	0.8	0.9
	HOT + SR	Mean	4.6	4.8	4.8	80	71	68	14.7	14.5	14.4	6.3	6.1	6.4
		SD	1.1	1.2	1.2	13	11	9	2.1	2.2	2.1	0.9	0.9	0.9
Static hose hold	SR	Mean	-	-	-	66	59	59	12.7	12.7	12.6	5.0	5.1	5.0
		SD	-	-	-	8	6	6	1.3	1.4	1.4	0.8	0.8	0.9
	HOT + SR	Mean	-	-	-	75	64	60	13.4	13.2	13.4	6.1	6.0	6.2
		SD	-	-	-	14	12	9	3.2	2.5	2.5	0.8	0.8	0.7

Work performance is reported in meters, apart from Rake which is reported in m^2^. RPE, rating of perceived exertion.

**Table 4:** Mean core temperature, hydration, and motivation for the SR and HOT + SR condition.

	Day					
	1		2		3	
	SR	HOT + SR	SR	HOT + SR	SR	HOT + SR
Core Temperature						
Daily core temperature (°C)	37.61 ± 0.21	37.85 ± 0.23	37.47 ± 0.29	37.66 ± 0.28	37.46 ± 0.25	37.58 ± 0.27
Hydration						
Urine specific gravity	1.015 ± 0.006	1.012 ± 0.008	1.011 ± 0.004	1.011 ± 0.008	1.011 ± 0.005	1.009 ± 0.007
Fluid in volume (mL) per circuit	1395 ± 344	2108 ± 367	952 ± 349	1443 ± 228	823 ± 602	1523 ± 318
Urine out volume (mL) per circuit	676 ± 293	609 ± 405	671 ± 316	651 ± 255	602 ± 298	681 ± 278
Motivation						
Effort sensation per circuit (%)	76 ± 11	81 ± 12	75 ± 11	77 ± 12	76 ± 9	76 ± 11

All data reported as means ± SD.
